# Learn from international recommendations and experiences of countries that have successfully implemented monoclonal antibody prophylaxis for prevention of RSV infection

**DOI:** 10.1186/s13052-025-01844-9

**Published:** 2025-02-04

**Authors:** Sara Manti, Eugenio Baraldi

**Affiliations:** 1 Department of Human Pathology of Adult and Childhood Gaetano Barresi, Pediatric Unit, University of Messina, Messina, Italy; 2 Department of Women’s and Children’s Health, Neonatal Intensive Care Unit, University of Padova, Padova, Italy; 3 Institute of Pediatric Research ‘Città della Speranza’, Padova, Italy

**Keywords:** Bronchiolitis, Implementation, Monoclonal antibodies, Nirsevimab, Prevention, Recommendations, Respiratory syncytial virus

## Abstract

Respiratory syncytial virus (RSV)-mediated bronchiolitis causes a significant global health burden. Despite this, for several years, the only approved agent for RSV prophylaxis was the anti-RSV monoclonal antibody Palivizumab, reserved for a small population of infants at high risk of developing severe RSV disease. Recently, the availability and approval of nirsevimab to immunize all infants against RSV infection since their first RSV season represented a crucial paradigm shift in RSV prevention. Nirsevimab has been shown to be safe and effective (> 80%) against RSV lower respiratory tract infections (LRTIs) in all infants and children at their first season of RSV. Surveillance studies have demonstrated 90% effectiveness in reducing all-cause hospitalizations, all-cause LRTI hospitalizations, RSV-related LRTI hospitalizations, and severe RSV-related LRTIs. Moreover, the consistency and reproducibility of the beneficial outcomes coming from the prophylaxis with nirsevimab highlights its potential to deliver substantial health benefits, positioning monoclonal antibody administration as a cornerstone in the fight against RSV-related morbidity and mortality. Implementing immunization strategies for infants and children is crucial to align the international experiences and guarantee universal protection. This review provided an updated overview of the monoclonal antibody strategy for preventing RSV infection.

## Introduction

Respiratory syncytial virus (RSV)-mediated bronchiolitis causes a significant global health burden, especially among infants within 5 years of age, with higher peaks during the winter months. It has been annually estimated that RSV-caused infections are approximately 33 million, with 3.2 million hospitalizations and 120,000 deaths, with a dramatic increase in the intensity of care for infants with bronchiolitis as assessed by increased intensive care admissions worldwide in the last years [1–3]. Moreover, the effects of RSV infection are long-lasting, beyond the acute infection, since the virus shows long-term respiratory consequences as it has been involved in the onset of recurrent wheezing and asthma [4, 5]. Consequently, considering its epidemiologic impact, the lack of a specific treatment, and its potential role in chronic lung diseases, reducing the global burden of RSV-related illness is considered a global health priority, and developing prevention strategies is a critical global priority [6]. The monoclonal antibody (mAb) palivizumab, produced by recombinant DNA technology and targeting the fusion (F) protein of the virus, represented the first approach for preventing RSV infections, and has been used in Italy since 1988 [7]. However, its administration is reserved for specific populations considered at high risk for severe RSV infection and complications and including premature newborns, infants diagnosed with pulmonary diseases, hemodynamically significant congenital heart diseases, cystic fibrosis, Down syndrome, congenital diaphragmatic hernia, neuromuscular diseases and immunodeficiency [8–10]. However, the abovementioned cluster of populations constitutes only 4–6% of the pediatric population potentially affected by RSV since 70% and 90% of children hospitalized due to RSV are neonates born healthy and at term, thus, ineligible for palivizumab prophylaxis [7, 11].

In addition to its restricted therapeutic indication, palivizumab is expensive, making it less accessible, and has short half-life, requiring monthly administration, affecting its impact in preventing RSV infection [7].

Aiming to overcome the limits of palivizumab, new long-acting mAbs are being developed to offer an alternative preventive approach to RSV infection. In this regard, a newer mAb, Nirsevimab, has been recently approved by the European Medicines Agency (EMA) [12] and Food Drug Administration (FDA) https://www.fda.gov/news-events/press-announcements/fda-approves-new-drug-prevent-rsv-babies-and-toddlers. Nirsevimab, a potent IgG1 neutralizing mAb, is derived from human B-cells and targets the conserved epitope on the fusion (F) protein. It offers immediate protection long-lasting of 5 months, covering the entire RSV season with a single intramuscular dose, to all newborns and infants, including healthy and at-term babies, thus overcoming the limit of palivizumab and revealing an appealing candidate for universal RSV prophylaxis. Specifically, the EMA approval of nirsevimab was based on three pivotal studies: study 3 [13], MELODY [14] and MEDLEY [15].

In the MELODY study, a multicenter, phase 3, placebo-controlled trial, enrolling 1490 healthy infants with gestational age ≥ 35 weeks, randomly assigned to receive a single dose of nirsevimab or placebo, authors reported that the incidence of RSV-associated lower respiratory tract infections (LRTIs) was significantly lower in the treated group compared to the placebo group; with an efficacy of 74.5% (95% confidence interval [CI], 49.6 to 87.1; *p* < 0.001) for nirsevimab [14]. Successively, in a phase 2b, authors demonstrated the efficacy of nirsevimab in preventing RSV infection also in healthy preterm newborns (29–35 weeks gestational age) [13]. Later, the MEDLEY study, a phase II/III, randomised, double-blind, palivizumab-controlled trial, aimed to assess safety and tolerability for nirsevimab in 925 preterm infants (≤ 35 gestational weeks) at high-risk eligible to receive palivizumab, and showed a similar safety profile to that of palivizumab [15].

Given its favourable efficacy and safety profile, and following EMA and FDA approval, several countries have included nirsevimab as universal prophylaxis into their national prevention plans. Herein, we summarized the international recommendations and experiences of countries that have successfully implemented pediatric prevention strategies against RSV, also aiming to contribute to homogeneously maximizing the adoption of nirsevimab in all Italian regions.

### Experience from United States of America

On June 8th, 2023, FDA Antimicrobial Drugs Advisory Committee (AMDAC) approved nirsevimab for the prevention of RSV LTRIs in all newborns and infants born during or entering their first RSV season and in newborns and infants with high risk for developing severe RSV infection, such as preterm babies (< 29 gestational age), newborns with hemodynamically significant heart diseases and or chronic lung diseases at their first and second epidemic season (up to 24 months of age) [16]. Accordingly, Centers for Disease Control and Prevention (CDC), Advisory Committee on Immunization Practices (ACIP), and American Academy of Pediatrics (AAP) recommended to include nirservimab in the official immunization schedule for children and infants. Also, AAP recommended a switch to nirsevimab for infants eligible for palivizumab [17, 18]. Supporting this recommendation, the New Vaccine Surveillance Network evaluated nirsevimab effectiveness against RSV-associated hospitalization among infants in their first RSV season, between October 2023 and February 2024. Authors reported that among the enrolled infants (*n* = 699) aged less than 8 months of age and hospitalized with acute respiratory illness, 59 (8%) received nirsevimab ≥ 7 days before symptom onset. Nirsevimab effectiveness was 90% against RSV-associated hospitalization with a median time from receipt to symptom onset of 45 days [19].

### Experience from France

On July 19th, 2023, the French National Authority for Health (HAS) approved the reimbursement of nirsevimab [20]. On September 15th, 2023, France was among the first countries that started a national immunization campaign [21].

Recommendations for the 2023–2024 winter season in France is to administrate nirsevimab to all infants under 12 months of age, also including newborns with well-known risk factors for severe RSV infection, at the start of the epidemic season. Due to the high adherence rates in France, nirsevimab was allocated for the immunization of newborns in maternity wards before discharge and for newborns under one month old in hospital wards starting on September [21].

By performing a case-control study, authors investigated the effectiveness of nirsevimab in preventing cases of severe RSV-mediated bronchiolitis and hospitalised in Pediatric Intensive Care Unit (PICU) in France. Healthy infants aged less than 1 month and infants with comorbidities (e.g., cardiac, pulmonary, renal, liver, neuromuscular or metabolic pathologies, cancer, immunodepression and diabetes) aged less than 5 months were included in the study. 238 cases and 50 controls enrolled from 20 PICUs were included in the final analysis, attesting the nirsevimab effectiveness at 75.9% (48.5–88.7) in the main analysis, and 80.6% (61.6–90.3) and 80.4% (61.7–89.9) in two sensitivity analyses in reducing severe RSV-mediated bronchiolitis [21]. In a multicentre, clinical trial (Hospitalized RSV Monoclonal Antibody Prevention (HARMONIE) study, also involving Germany and the United Kingdom, and enrolling 8058 infants, randomly assigned to receive nirsevimab (4037 infants) or standard care (4021 infants), only 0.3% (num. 11 infants) in the nirsevimab group were hospitalized for RSV-associated LRTIs compared to 1.5% in the standard-care group (60 infants). These findings showed nirsevimab efficacy of 83.2% (95% confidence interval [CI], 67.8 to 92.0; *p* < 0.001). Regarding the incidence of very severe LRTIs, the latter occurred in 0.1% infants with RSV infection (5 infants) in the nirsevimab group compared to 0.5% in the standard-care group (19 infants), attesting the nirsevimab efficacy of 75.7% (95% CI, 32.8 to 92.9; *p* = 0.004). Lastly, the efficacy of nirsevimab against hospitalization for RSV-associated LTRIs was 89.6% in France. Regarding the treatment-related adverse events, the latter was reported in 2.1% of infants in the nirsevimab group (86 infants) [22].

To assess the impact of nirsevimab on the bronchiolitis epidemic, researchers developed a mathematical model of RSV transmission among different age groups, based on the number of treatment doses supplied to maternity hospitals and pharmacies, virological surveillance data for the period from 2017 to February 2024, as well as serological data. Authors showed that the administration of nirsevimab would prevent 5.800 hospitalizations for RSV bronchiolitis (95% CI: 3.700–7.800), of which 4.200 among children aged 0 to 2 months. This finding corresponded to a 23% (16–30%) reduction in the total number of hospitalizations for RSV bronchiolitis compared with the non-administration scenario. In the baseline scenario, with 215.000 doses administered by February 1st, 2024, the efficacy of nirsevimab against hospitalizations for RSV bronchiolitis was estimated to be 73% (61–84%), corresponding to one hospitalization prevented for every 39 (26–54) doses administered [23].

More recently, following the implementation of nirsevimab in France, authors found a 52.7% (95% CI: 46.4–58.9) decrease in all-cause bronchiolitis in children aged less than 3 months of age with the lowest number of bronchiolitis cases [24].

In line with these findings, in a prospective, multicenter, matched case-control study enrolling 1035 infants (690 subjects were case patients and 345 were matched controls), authors reported that the effectiveness of nirsevimab against hospitalization for RSV-caused bronchiolitis was 83.0%. Also, the effectiveness of nirsevimab therapy against RSV-associated bronchiolitis resulting in critical care was 69.6% and against RSV-associated bronchiolitis requiring ventilatory support was 67.2% [25].

### Experience from Spain

In Spain, nirsevimab has been adopted since the 2023–2024 epidemic season and destinated to all newborns up to 6 months of age at their first RSV season and newborns up to 24 months of age with risk factors for severe RSV infection at their second RSV season, with inclusion of nirservimab in the national vaccination program [26].

The Spanish experience has been attested on 10,259 children, 6–24 months aged, without and with risk factors for RSV infection, enrolled during September 2023 and March 2024 [27]. Authors reported high coverage during the immunization campaign with nirsevimab: 95.4% in infants born in season, 89.9% in those born out of season, and 97% in newborns at high-risk for severe RSV infection [27]. Nirsevimab effectiveness was 66.2% (56–73.7%) against all-cause hospitalizations, 69.2% (55.9 − 78%) against all-cause LRTI hospitalizations, 82% (65.6–90.2%) against RSV-related LRTI hospitalizations, and 86.9% (69.1–94.2%) against severe RSV-related LRTI requiring oxygen support. The number needed to immunise to avoid one RSV-related LRTI hospitalization was 25 (IQR 24–32). No severe adverse events related to nirsevimab adminsitration were registered [27].

High coverage rates (up to 92%) and effectiveness in RSV-related hospitalization (up to 89%) have also been reported in Valencia, Murcia, Valladolid, Catalogna, and Navarre [28, 29].

Recently, the National Immunization Technical Advisory Group (NITAG) and Ministerio de Sanidad have confirmed the recommendation to use nirsevimab as a single strategy for protecting all infants and children from RSV in the 2024–2025 season. This decision was based on the high coverage during the immunization campaign with nirsevimab (92.4% in infants born in season and 84.8% in those born out of season), the good safety profile observed, and the remarkable reduction in hospitalizations caused by RSV among children under 1 year compared to the previous season (90% in infants born in season and 89% in those born out of season), and the evidence that that in children (1–4 years aged) not receiving prophylaxis, the rate of RSV-related hospitalization did not change compared to the previous epidemic seasons. An official paper statement summarizing the recommendations for nirsevimab and maternal immunization is expected from NITAG for the 2025–2026 season [30].

### Experience from Italy

To date, Valle d’Aosta has been the only Italian region to start universal prophylaxis with nirsevimab for newborns and infants born at their first 2023–2024 epidemic season. 556 infants, born from May 1st, 2023 to February 15th, 2024 were recruited. 16/556 subjects were affected by prematurity or other comorbidities; thus, they received prophylaxis with palivizumab according to the current guidelines of the Italian Society of Neonatology (SIN) [10]. 3/556 were excluded from the study due to non-residency in the region. Overall, 537 patients were included in the study and of them, 369/537 were treated with nirsevimab, with a participation rate of 69%, suggesting a significant level of adherence to nirsevimab prophylaxis among eligible neonates. When compared to 7% in the 2022–2023 epidemic season, the hospitalization for RSV-caused bronchiolitis in 2023–2024 was 3.2% (*p* < 0.001). Moreover, the risk of hospitalization for bronchiolitis in the cohort of infants who did not adhere to the prophylaxis was 8.3% compared to 0% in the cohort that received nirsevimab (*p* < 0.001) [31]. Regarding safety data, authors reported only mild side effects, generally manifested within 48 h post-treatment and lasting 1–2 days. The most common side effects were fever (6.5%), local reactions at the injection site (4%), and consolable crying (0.4%). No patientsneeded a medical visit [31].

On February 2024, taking into account the international and national experience as well as effectiveness and safety data, the Italian Society of Infectious and Tropical Diseases (SIMIT), the Italian Society of Hygiene, Preventive Medicine and Public Health (SItI), SIN, the Italian Society of Pediatrics (SIP), the Italian Federation of Primary Care Pediatricians (FIMP), and the Italian Federation of Family Doctors (FIMG) recognized nirservimab as a potential and universal prevention weapon against RSV infection and designed for all newborns and infants born at their first epidemic season, which should be included in the national vaccination program, like a vaccine [32, 33]. In this regard, the update of Italian guidelines on managing bronchiolitis in infants already supported the use of maternal vaccines and long-acting mAbs in all infants to prevent RSV infection and its short- and long-term consequences [34].

Accordingly, on March 27th, 2024, the Ministry of Health announced that the National Vaccine Prevention Plan (PNPV) 2023–2025 should be adapted to the current epidemiological situation and include mAbs for preventing infectious diseases, such as nirservimab, to counteract the RSV infection since the next epidemic season 2024/2025 [35]. Following this statement, several Italian countries will start from October, and nirsevimab will be recommended to all neonates born at their first RSV epidemic season (from October 1st to March 31st ) from birth and up to 13 months of age, also including subjects at high-risk for severe RSV infection, such as preterm babies and/or comorbidities. Specifically, nirsevimab should be administered to babies born in-season within 24–48 h after birth, preferably at the birth centre before discharge. For babies born from April 1st to September 30th, they will have to be immunized by primary care paediatricians before the start of the epidemic season and until March 31st, and within the 13th month of age (Table 1) [36, 37].

**Table 1:**
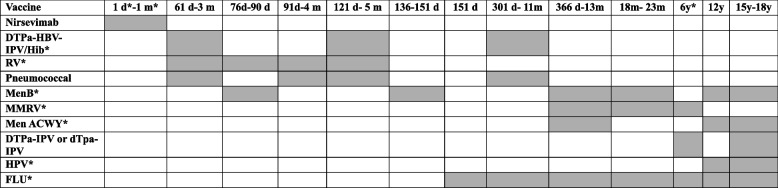
Sicily vaccine prevention plan (PNPV) 2024 – from 1 day to 18 years

*D day, *M* month, *Y* year, *DTPa-HBV-IPV/Hib *Diphtheria-tetanus-acellular pertussis-hepatitis B recombinant (adsorbed)-inactivated-Hepatitis B virus- poliomyelitis-adsorbed conjugated Haemophilus influenzae type b, *RV* Rotavirus, *MenB* meningococcal group B bacteria, *MMRV* measles, mumps, rubella, varicella, *Men ACWY* meningococcal group ACWY, *HPV*, Papilloma virus, *FLU* Influenza virus

### Experiences from other countries

#### In Germany

Starting from the 2024–2025 season, the German Standing Commission on Vaccination (STIKO) recommends prophylaxis with nirsevimab, especially for newborns and infants at risk for developing severe RSV infection, such as premature infants or babies with heart defects. Ideally, newborns born during the RSV season -between October and March- should receive the nirservimab immediately after birth and before hospital discharge, while babies born outside the RSV season- between April and September- should receive nirsevimab in the fall but before the beginning of their first RSV season [38]. This recommendation aims to prevent the incidence of severe RSV infection, and, consequently, reduce hospitalization and death rates [38]. Moreover, STIKO concludes that the current evidence for maternal vaccination needs to be revised to make a recommendation due to the small study population and the need for more data supporting its effectiveness and safety [38].

#### In the United Kingdom

The Joint Committee on Vaccination and Immunisation (JCVI) recommend maternal vaccination to protect infants from RSV infection. Starting from September 1st, 2024, all pregnant women at 28 weeks gestational age should be offered a single dose of the RSV vaccine [39].

High-risk infants and children should be offered a monoclonal antibody immunisation regardless of whether their mother received an RSV vaccine in pregnancy.

No official recommendation or guidance is provided regarding nirservimab in healthy infants at their first RSV season. Considering its extended half-life, high efficacy and lower price, the JCVI discussed the possibility of switching to nirsevimab infants eligible for palivizumab, but, to date, specific advice has not been provided [39].

#### In Luxembourg

In Luxembourg, the national campaign started in the autumn of 2023 and planned to administer one-dose nirsevimab prophylaxis to all babies born in 2023 and included a catch-up immunization for children younger than 2 years of age and at high risk of serious illness at their second RSV season. The national immunization included 84% newborns (1.277 doses/1.524 births) in 2023 and resulted in a drop in hospitalization of 69% among infants younger than 6 months of age and a decrease of 38% among children under the age of 5 years. Moreover, when compared to the previous season, data revealed a decrease in the severity of RSV infection since a reduction in length of stay (5 days vs. 3 days) as well as in ICU admission (28 vs. 9 infants) were also reported [40].

#### In Belgium

The Superior Health Council (SHC) supported nirsevimab for the prevention of RSV disease for infants younger than 1 year of age and entering the first RSV season, regardless of the presence of comorbidities or risk factors for severe RSV infection. Nirsevimab should also be administered to all babies born from unvaccinated mothers or born prematurely (< 30 weeks of gestational age) or within the two weeks following the maternal vaccine administration. For most infants, administering both products is not indicated; however, administration of nirsevimab to babies born from vaccinated mothers could be considered in infants with increased risk for severe RSV disease, infants born from mothers vaccinated at the end of the season, infants whom mothers have an inadequate immune response to vaccination (immunocompromised status) or decreased transplacental antibody transfer (people living with HIV infection or membrane diseases), infants with cardiopulmonary bypass or receiving blood transfusion, leading to a reduced serum maternal antibodies levels.

The SHC extended the prevention to children 1 and 2 years at their second RSV season, whether risk factors for severe infections are still present.

Moreover, whether for winter 2023–2024, the SHC recommended to confirm palivizumab for infants at high risk of RSV, for season 2024–2025, the switch from palivizumab to nirservimab is expected [41].

#### In Netherlands

The Health Council of the Netherlands (HCN) recommends that immunization with nirsevimab should be administered to all children before or during the RSV season, immediately after the birth and within 2 weeks at the latest. For babies born after the RSV season, nirsevimab should be administered before the start of their first RSV season [42]. Moreover, HCN expressed its preference for the use of nirsevimab rather than maternal vaccination against RSV since the seasonal timing of offering allows for the protection of a larger population of children, including preterm babies, with a higher safety profile than maternal vaccination [42]. Lastly, HCN pushes for protection against RSV to be included in the NPV as soon as possible [42].

#### In Sweden

In Sweden, nirsevimab is recommended in all newborns and children at their first season, and it is also preferred to maternal vaccination since the latter alone cannot protect preterm infants (< 32 weeks) and babies with other risk factors for developing severe RSV infection [43].

#### In Finland

The Council for Choices in Health Care (COHERE) has approved nirservimab in the prevention of LRTIs caused by RSV for healthy children younger than 3 months of age and during the epidemic period and in children at higher risk of RSV infection and hospitalization [44].

#### In Canada

Canada’s National Advisory Committee on Immunizations (NACI) has issued a preferential recommendation for a universal RSV immunization program with nirsevimab for (1) all newborns born during or enter their first RSV season and who are at increased risk of severe RSV disease and/or who have complex access to care and are influenced by social determinants; and (2) all infants younger than 8 months entering or during their first RSV season. Lastly, NACI does not recommend a specific vaccination schedule for the RSVpreF vaccine. Instead, it suggests that maternal vaccination should be considered as an individual decision for the pregnant woman [45].

#### In Brazil

In Brazil, nirsevimab has been included in the official immunization schedule for children and infants. The Brazilian Society of Immunization (SBIm) recommended nirsevimab to prevent RSV in all newborns during the first RSV season and in high-risk newborns during the second season [46].

#### In Australia

The Australian Technical Advisory Group on Immunization (ATAGI) recommends administering nirsevimab at birth or before the start of the first RSV season. It also recommends administering nirsevimab in children up to 24 months before the second RSV season, with dosing adjusted to efficacy [47].

#### In Japan

The Ministery of Health, Labour and Welfare (MHLW) of Japan supported nirsevimab in preventing RSV infection and included the mAb in the National Plan of Immunization (NPI) [48].

#### In Madeira

On the island of Madeira, the immunization campaign with nirsevimab achieved high coverage rates, around 95.3% in children born in season and 92.5% in children born out of season [49].

#### In Chile

From March 2024, the Comité Asesor en Vacunas y Estrategias de Vacunación (CAVEI) recommended to administer nirsevimab to all newborns and infants at their first season of RSV and a second dose also in children at high-risk for severe infection at their second RSV season. Moreover, the CAVEI suggested to include nirsevimab in the programmatic vaccines schedule [50].

## Conclusions

RSV is the leading cause of outpatient medical care and hospitalization in all newborns worldwide [2, 51]. To predict how many and which newborns will develop a severe RSV infection is not possible, and until sometime ago, weapons to protect healthy and at-term newborns, constituting the pediatric population more commonly developing RSV infection, were not available, since they were ineligible for palivizumab prophylaxis.

Thanks to the availability of new mAbs, it is possible to guarantee protection to all infants and children since their first RSV season. Moreover, it is also reasonable to hypothesize that, by preventing the acute and short-term consequences of RSV infection, it is possible to prevent the long-term respiratory consequences, such as wheezing and asthma, in babies previously affected by RSV. As a result, the World Health Organization (WHO) strongly recommends considering the new mAbs as a public health intervention, with the potential to be included in vaccination schedules [52]. Nevertheless, considerable variability among countries has been documented in terms of geographical settings, immunization campaigns, availability for nirsevimab, and eligibility criteria for passive immunization (Table 2). Despite these limits, nirsevimab has been shown in clinical trials to be safe and effective (> 80%) against RSV LRTIs in all infants and children at their first season of RSV. Surveillance studies have demonstrated 90% effectiveness in reducing all-cause hospitalizations, all-cause LRTI hospitalizations, RSV-related LRTI hospitalizations, and severe RSV-related LRTIs. Thus, the consistency and reproducibility of the beneficial outcomes from the prophylaxis with nirsevimab highlights its potential to deliver substantial healthy benefits, positioning mAb administration as a cornerstone in the fight against RSV-related morbidity and mortality. Implementing immunisation strategies for infants and children is crucial to align the international experiences and guarantee universal protection.

**Table 2:** Summary of international and national recommendations for nirservimab prescription

Country	Recommended by	Epidemic season	Recommended in	Administered where	Switch from PVZ to nirsevimab	Included in the immunization calendar
USA	CDC, ACIP, AAP	2023–2024	1. All newborns and infants < 8 months born during or entering their first RSV season 2. In newborns and infants with high risk for developing severe RSV infection at their first and second epidemic season (up to 24 months of age)	Hospital inside Hospital outside	Yes	Yes
France	FHAS	2023–2024	All infants under 12 months of age, also including newborns with well-known risk factor for severe RSV infection, at the start of the epidemic season.	Hospital inside Hospital outside	Not stated	Yes
Spain	Ministerio de Sanidad NITAG	2023–2024	1. All newborns up to 6 months of age at their first RSV season 2. Newborns with risk factors for severe RSV infection at their second RSV season, up to 24 months of age.	Hospital inside Hospital outside	Not stated	Yes
Italy	Ministry of Health SItI SIMIT SIN SIP FIMP FIMMG	2024–2025	All neonates born at their first RSV epidemic season from birth and up to the 13 months of age, also including subjects at high risk for severe RSV infection.	Hospital inside Hospital outside	Not stated	Yes, in the PNPV 2023–2025
Germany	STIKO	2024–2025	Healthy newborns and infants a risk for developing severe RSV infection born during and outside their first epidemic RSV season.	Hospital inside Hospital outside	Not stated	No
UK	JCVI	2023–2024	Not specified. UK recommended that PVZ was replaced by nirsevimab for the eligible infants.	Hospital inside Hospital outside	Not stated	No
Luxembourg	SCID	2023–2024	1. All newborns < 6 months at their first RSV season 2. Newborns with risk factors for severe RSV infection at their second RSV season, up to 24 months of age.	Not stated	Not stated	No
Belgium	SHC	2023–2024	1. All infants younger than 1 year of age entering at the first RSV season 2. All babies born from unvaccinated mothers or born prematurely (< 30 weeks of gestational age) or within the two weeks following the maternal vaccine administration. 3. Infants a risk for developing severe RSV infection at their secondo RSV season, up to 24 months of age.	Hospital inside Hospital outside	Yes	Not stated
Netherlands	HCN		1. All children before or during the RSV season, immediately after the birth and within 2 weeks at the latest. 2. Babies born after the RSV season, nirsevimab should be administered before the start of their first RSV season	Not stated	Not stated	Not yet but it is planned
Sweden	TRR	2024–2025	All infants and children at their first season	Not stated	Not stated	No
Finland	COHERE	2024–2025	1. Healthy newborns and infants younger than 3 months at their first RSV season 2. All children at high risk for severe RSV infection	Not stated	Not stated	No
Canada	NACI	2024–2025	1. All newborns < 8 months born during or enter their first RSV season and who are at increased risk of severe RSV disease and/or who have complex access to care and are influenced by social determinants	Not stated	Not stated	No
Brazil	SBIm	2024–2025	1. All newborns during the first RSV season 2. In high-risk newborns during the second season	Not stated	Not stated	No
Australia	ATAGI	2024–2025	All newborns during their first and second RSV season, up to 24 months of age.	Not stated	Not stated	Not stated
Japan	MHLW	Not stated	To define	Not stated	Not stated	Not yet but it is planned
Madeira	DRS	2023–2024	All newborns during their first RSV season	Not stated	Not stated	Yes
Chile	CAVEI	March 2024	1. All newborns during the first RSV season 2. In high-risk newborns during the second season	Not stated	Not stated	Not stated

*USA* United States of America, *CDC* Centers for Disease Control and Prevention, *ACIP* Advisory Committee on Immunization Practices, *AAP* American Academy of paediatrics, *PVZ* Palivizumab, *RSV* Respiratory Syncytial virus, *FHAS* French National Authority for Health, *NITAG* National Immunization Technical Advisory Group, *SItI* Italian Society of Hygiene, Preventive Medicine and Public Health, *SIMIT* Italian Society of Infectious and Tropical Diseases, *SIN* Italian Society of Neonatology, *SIP* Italian Society of Paediatrics, *FIMP* Italian Federation of Pediatricians, *FIMMG* Italian Federation of General Practitioners, *STIKO* German Standing Commission on Vaccination, *UK* United Kingdom, *JCVI* Joint Committee on Vaccination and Immunisation, *SCID* Superior Council for Infectious Diseases, *SHC* Superior Health Council, *HCN* Health Council of the Netherlands, *TRR* Tjänsteutbud Rådets Rekommendation, *COHERE* Council for Choices in Health Care, *NACI* National Advisory Committee on Immunizations, *SBIm* Brazilian Society of Immunization, *ATAGI* Australian Technical Advisory Group on Immunization, *MHLW* Ministery of Health, Labour and Welfare, *DRS* Direção Regional da Saúde, *CAVEI* Comité Asesor en Vacunas y Estrategias de Vacunación

## Data Availability

Not applicable.

## Abbreviations

AAP: American Academy of pediatrics
ACIP: Advisory Committee on Immunization Practices
AMDAC: Antimicrobial Drugs Advisory Committee
ATAGI: Australian Technical Advisory Group on Immunization
CAVEI: Comité Asesor en Vacunas y Estrategias de Vacunación
CDC: Centers for Disease Control and Prevention
COHERE: Council for Choices in Health Care
EMA: European Medicines Agency
FDA: Food Drug Adminsitration
FIMG: Italian Federation of Family Doctors
FIMP: Italian Federation of Primary Care Pediatricians
HAS: National Authority for Health
HCN: Health Council of the Netherlands
JCVI: Joint Committee on Vaccination and Immunisation
LTRIs: lower respiratory tract infections
mAb: monoclonal antibody
MHLW: Ministery of Health, Labour and Welfare
NACI: National Advisory Committee on Immunizations
NITAG: National Immunization Technical Advisory Group
PICU: Pediatric Intensive Care Unit
PNI: National Plan of Immunization
PNPV: National Vaccine Prevention Plan
RSV: Respiratory syncytial virus
SBIm: Brazilian Society of Immunization
SHC: Superior Health Council
SIMIT: Italian Society of Infectious and Tropical Diseases
SIN: Italian Society of Neonatology
SIP: Italian Society of Pediatrics
SItI: Preventive Medicine and Public Health
STIKO: German Standing Commission on Vaccination
