# Correction: Learn from international recommendations and experiences of countries that have successfully implemented monoclonal antibody prophylaxis for prevention of RSV infection

**DOI:** 10.1186/s13052-025-01901-3

**Published:** 2025-02-13

**Authors:** Sara Manti, Eugenio Baraldi

**Affiliations:** 1 Department of Human Pathology of Adult and Childhood Gaetano Barresi, Pediatric Unit, University of Messina, Messina, Italy; 2 Neonatal Intensive Care Unit, Department of Woman’s and Child’s Health, University Hospital of Padova, Padua, Italy; 3 Institute of Pediatric Research ’Città della Speranza’, Padova, Italy; 4 Respiratory Syncytial Virus Network (RESVINET) Foundation, Zeist, the Netherlands

**Correction to: Italian Journal of Pediatrics (2025) 51:26**

10.1186/s13052-025-01844-9

The authors wish to note the following amendments to the affiliations affecting co-author, Eugenio Baraldi:

^1^Department of Human Pathology of Adult and Childhood Gaetano Barresi, Pediatric Unit, University of Messina, Messina, Italy

^2^Neonatal Intensive Care Unit, Department of Woman's and Child's Health, University Hospital of Padova, Padua, Italy

^3^Institute of Pediatric Research'Città della Speranza', Padova, Italy

^4^Respiratory Syncytial Virus Network (RESVINET) Foundation, Zeist, the Netherlands

